# Oligomeric forms of amyloid-β protein in plasma as a potential blood-based biomarker for Alzheimer’s disease

**DOI:** 10.1186/s13195-017-0324-0

**Published:** 2017-12-15

**Authors:** Min Jeong Wang, SangHak Yi, Jee-young Han, So Young Park, Jae-Won Jang, In Kook Chun, Sang Eun Kim, Byoung Sub Lee, Gwang Je Kim, Ji Sun Yu, Kuntaek Lim, Sung Min Kang, Young Ho Park, Young Chul Youn, Seong Soo A. An, SangYun Kim

**Affiliations:** 1 Department of Neurology, Seoul National University Bundang Hospital and Seoul National University College of Medicine, 82, Gumi-ro 173, Bundang-gu, Seongnam-si, Gyeonggi-do 463-707 Republic of Korea; 2 Department of Neurology, Kangwon National University Hospital, Chuncheon-si, Republic of Korea; 3 Department of Nuclear Medicine, Kangwon National University Hospital and School of Medicine, Kangwon National University, Chuncheon-si, Republic of Korea; 4 Department of Nuclear Medicine, Seoul National University Bundang Hospital and Seoul National University College of Medicine, Seongnam-si, Republic of Korea; 5 Department of Research and Development, PeopleBio, Inc, Seongnam-si, Republic of Korea; 6 Department of Neurology, Chung-Ang University Hospital, Seoul, Republic of Korea; 7 Department of Bionano Technology, Gachon Medical Research Institute, Gachon University, 1342, Sengnamdaero, Sujeong-gu, Seongnam-si, Gyeonggi-do 461-701 Republic of Korea

**Keywords:** Amyloid-β protein, Oligomer, Alzheimer’s disease, Biomarker

## Abstract

**Background:**

Soluble amyloid-β (Aβ) oligomers are the major toxic substances associated with the pathology of Alzheimer’s disease (AD). The ability to measure Aβ oligomer levels in the blood would provide simple and minimally invasive tools for AD diagnostics. In the present study, the recently developed Multimer Detection System (MDS) for AD, a new enzyme-linked immunosorbent assay for measuring Aβ oligomers selectively, was used to detect Aβ oligomers in the plasma of patients with AD and healthy control individuals.

**Methods:**

Twenty-four patients with AD and 37 cognitively normal control individuals underwent extensive clinical evaluations as follows: blood sampling; detailed neuropsychological tests; brain magnetic resonance imaging; cerebrospinal fluid (CSF) measurement of Aβ42, phosphorylated tau protein (pTau), and total tau protein (tTau); and ^11^C-Pittsburgh compound B (PIB) positron emission tomography. Pearson’s correlation analyses between the estimations of Aβ oligomer levels by MDS and other conventional AD biomarkers (CSF Aβ_42_, pTau, and tTau, as well as PIB standardized uptake value ratio [PIB SUVR]) were conducted. ROC analyses were used to compare the diagnostic performance of each biomarker.

**Results:**

The plasma levels of Aβ oligomers by MDS were higher in patients with AD than in normal control individuals, and they correlated well with conventional AD biomarkers (levels of Aβ oligomers by MDS vs. CSF Aβ_42_, *r* = −0.443; PIB SUVR, *r* = 0.430; CSF pTau, *r* = 0.530; CSF tTau, *r* = 0.604). The sensitivity and specificity of detecting plasma Aβ oligomers by MDS for differentiating AD from the normal controls were 78.3% and 86.5%, respectively. The AUC for plasma Aβ oligomers by MDS was 0.844, which was not significantly different from the AUC of other biomarkers (*p* = 0.250).

**Conclusions:**

Plasma levels of Aβ oligomers could be assessed using MDS, which might be a simple, noninvasive, and accessible assay for evaluating brain amyloid deposition related to AD pathology.

**Electronic supplementary material:**

The online version of this article (doi:10.1186/s13195-017-0324-0) contains supplementary material, which is available to authorized users.

## Background

Brain amyloidosis is a critical feature of Alzheimer’s disease (AD), and it was recently introduced as a diagnostic criterion [1]. Currently, prominent amyloid biomarkers of AD are amyloid-β 1–42 (Aβ_42_) levels in the cerebrospinal fluid (CSF) and amyloid positron emission tomography (PET) imaging [1]. Recently, these methods have been used widely in the clinical setting; however, they have several disadvantages, such as cost, invasiveness, and interlaboratory variability. Although simple and inexpensive blood-based biomarkers would be preferable for their safety and minimal invasiveness, no such biomarker having a direct association with the pathomechanism of AD has been developed thus far [2–4]. With accumulating evidence of soluble Aβ oligomers being the major toxic substances of AD pathology [5–8], efforts for measuring oligomers in plasma have been increasing [9–11]. Hence, the reliable measurement of Aβ oligomers in blood samples would present a noninvasive, inexpensive, and accessible method for making the AD diagnosis.

**Fig. 1:**
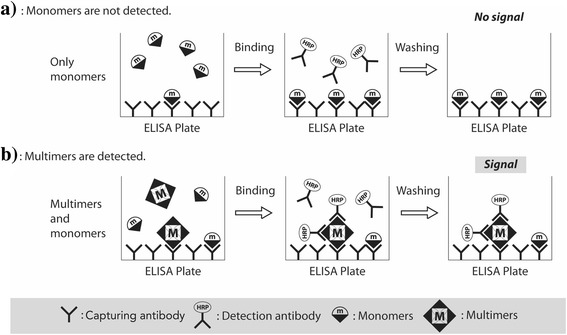
The Multimer Detection System concept. **a** Monomers are proteins with a single epitope that can be captured by an antibody (capturing antibody 6E10) attached to the surface of the plate. After the addition of a detection antibody (FF51-horseradish peroxidase [HRP]), monomer proteins cannot be detected, because the single epitope is already occupied. **b** Multimers with numerous epitopes can be detected by detection antibodies. The capturing and detection antibodies are different, but their epitopes overlap. *ELISA* Enzyme-linked immunosorbent assay

A promising enzyme-linked immunosorbent assay (ELISA)—the Multimer Detection System (MDS) for differentiating multimers from their cellular monomers—has been developed for quantifying various oligomers. MDS was originally designed to specifically detect prion oligomers in the blood from scrapie-infected animals for a scrapie blood test [12, 13]. MDS resembles a sandwich ELISA, as illustrated in Fig. 1. One specific and unique epitope existed in the Aβ monomer, and multiple copies of this epitope existed in the multimers. Hence, if epitope-overlapping antibodies toward the above unique epitope were used for capturing and detecting antibodies, binding to a specific and unique epitope would generate competition between these two antibodies. In other words, the monomer would be occupied by the capturing or detection antibody but not by both. Multiple copies of the above unique epitope in multimers would allow the binding of both the capturing and detection of antibodies, which would produce detectable signals from the detection antibody.

In this study, MDS was further developed to measure the levels of Aβ oligomers in plasma of patients with AD and cognitively normal control subjects (NC) using epitope-overlapping Aβ antibodies toward the N-terminus. The correlations between the levels of Aβ oligomers and other AD biomarkers, including CSF Aβ_42_, total tau protein (tTau), phosphorylated tau protein (pTau), and amyloid PET imaging, were analyzed. The results we present suggest the possibility of using MDS for measuring plasma Aβ oligomer levels as a simple blood-based biomarker test for making the AD diagnosis.

## Methods

### Subjects

Participants were enrolled at the Neurocognitive Behavior Center of the Seoul National University Bundang Hospital, as well as at the Department of Neurology of Chung-Ang University Hospital, Republic of Korea, from April 2012 to November 2014. All participants underwent clinical evaluations of detailed neuropsychological tests and brain magnetic resonance imaging (MRI) at baseline for the accurate diagnosis of AD and NC. The inclusion criteria for the AD group were as follows: (1) probable AD dementia according to the National Institute on Aging-Alzheimer’s Association criteria [1], (2) age between 50 and 90 years, (3) ≥ 6 years of education, (4) Clinical Dementia Rating (CDR) of 0.5–2 and CDR Sum of Boxes score ≥ 2.5, (5) modified Hachinski ischemic score ≤ 4, and (6) having a caregiver who knew the patient well. The inclusion criteria for the NC group were as follows: (1) age between 50 and 90 years, (2) subjects who did not have health factors influencing cognitive performance [14], (3) Mini Mental State Examination (MMSE) score > 1.0 SD below the mean for their age- and education-matched norm, (3) ≥ 6 years of education, and (4) Geriatric Depression Scale (short form) score ≤ 7. We excluded subjects if they had (1) major medical problems, (2) psychiatric problems, (3) a cardiac pacemaker, and/or (4) a history of substance abuse or dependence within the past 10 years. Ultimately, 24 patients with AD and 37 NC were enrolled in this study.

The study was approved by the institutional review board of the Seoul National University Bundang Hospital and Chung-Ang University Hospital [B-1202-145-003, B-0905-075-003, C2013142(1102), C2012048(743)]. Written informed consent was obtained from all patients (or their caregivers) who participated in the study.

### Blood sampling

Venous blood was collected in 10-ml sodium heparin-containing tubes (BD-367874; BD Biosciences, San Jose, CA, USA) and centrifuged at 850 × *g* for 30 minutes at room temperature. The allowed time delay between the collection and centrifugation was within 3 h, and the separation of plasma was performed within the allowed time. The plasma supernatant was aliquoted and stored in screw cap polypropylene tubes (0.5-ml Axygen tube, ST-050-SS; Corning Life Sciences, Tewksbury, MA, USA) at −80 °C until further analysis.

### MDS

#### Preparation of synthetic Aβ_42_

Lyophilized AggreSure Aβ_42_ peptide (AnaSpec, Fremont, CA, USA) was dissolved in 50 mM Tris/150 mM NaCl (pH 7.2) at a concentration of 1 mg/ml and then sonicated for 5 minutes. The homogenous peptide solution was further diluted with PBS containing Tween 20 (PBST; Sigma-Aldrich, St. Louis, MO, USA) to a desired concentration of 10 μg/ml. Solutions of diluted peptides were divided into aliquots and kept at −80 °C until further use.

#### MDS for Alzheimer’s disease

The basic detailed concept of the MDS technique for measuring oligomeric amyloid content by spiking the synthetic Aβ_42_ peptide level and the incubation time were reported recently [15]. Briefly, epitope-overlapping antibodies specific for the N-terminus of Aβ were used to capture and detect the Aβ antigen in its multimeric or oligomeric form. The mouse monoclonal antibody 6E10 for the epitope of Aβ_42_ residues 3–8 (BioLegend, San Diego, CA, USA) and the FF51-horseradish peroxidase (FF51-HRP) antibody (PeopleBio Inc., Seongnam-si, South Korea) for the epitope of Aβ residues 1–4 at the N-terminus were used to detect Aβ oligomers by the MDS assay. The epitopes for these antibodies overlapped at the N-terminus 3–4 of Aβ.

The wells of a 96-well black plate were coated with 3 μg/ml dilution of the 6E10 antibody in carbonate-bicarbonate buffer (Sigma-Aldrich) overnight at 4 °C for MDS preparation (Thermo Fisher Scientific, Waltham, MA, USA). The plates were blocked for 2 h with 0.4% Block Ace (100 μl) at room temperature. After the plate was washed thrice with PBS (Sigma-Aldrich), it was stored at 4 °C until use. Prior to the assay, aliquots of plasma samples were thawed at 37 °C for 15 minutes. Ten microliters of plasma, 4.04 μl of HBR-1 (a human antimouse antibody blocker; Scantibodies Laboratory, Santee, CA, USA), and PBST were mixed well and incubated in the presence of synthetic Aβ_42_ at 37 °C for 144 h.

The plasma sample mixture and the serially diluted recombinant Aβ standards were added to each well of the plate in a total volume of 100 μl. The plates were incubated at room temperature for 1 h. After three washes with Tris-buffered saline with Tween 20 (TBST), the FF51-HRP antibody in TBST containing 0.4% Block Ace was added to the wells, and the plate was incubated for 1 h at room temperature. To increase the sensitivity of detection, 100 μl/well of enhanced chemiluminescent substrate solution (Rockland Immunochemicals Inc., Limerick, PA, USA) was used, and the luminescence signal was detected and quantified using a VICTOR 3™ multispectrophotometer (PerkinElmer, Waltham, MA, USA).

### CSF study

CSF was collected following an updated version of the Korean consensus protocol of CSF AD biomarkers regarding preanalytical factors [16, 17]. In brief, a lumbar puncture was performed at the space between levels L3/L4 or L4/L5 in the morning (8:00 a.m.–12:00 p.m.). An experienced interventional radiologist performed the lumbar puncture using fluoroscopy with either Quincke (20-gauge) or “atraumatic” Whitacre (20- or 22-gauge) needles. A total of 10–15 ml of CSF was collected in a 15-ml centrifuge tube by free flow. Centrifugation at 2000 × *g* for 10 minutes at room temperature was done within 4 h after the lumbar puncture. CSF biomarkers of AD (Aβ_42_, pTau, and tTau) were analyzed using INNO-BIA AlzBio3 immunoassay kit-based reagents (Fujirebio Europe, Ghent, Belgium) [18].

### ^11^C-Pittsburgh compound B PET study

Participants underwent ^11^C-Pittsburgh compound B (PIB) PET. Static PET image acquisition for 20 minutes was performed 40 minutes after an intravenous bolus injection of PIB (659 ± 122 MBq) in each subject. Each PIB PET image was coregistered to T1-weighted MRI of each subject and spatially normalized to the T1-weighted brain MRI template. Then, MRI-based segmentation of cerebral gray and white matter was performed, and the mean standardized uptake value (SUV) was calculated in each brain region using modified automated anatomical labeling. The mean cortical PIB SUV ratio (PIB SUVR) was calculated as the mean uptake over voxels in the prefrontal, anterior cingulate, posterior cingulate, precuneus, lateral temporal, and parietal regions of interest for each subject, divided by the mean uptake over voxels in the cerebellar gray matter.

### Statistical analyses

Baseline characteristics were compared between various groups of data using unpaired *t* and chi-square tests. For variables with a nonnormal distribution (MMSE, CSF tTau/Aβ_42_ ratio, PIB SUVR, and MDS relative light units [RLU]), the Wilcoxon rank-sum test was used, and the median values were calculated. Pearson’s correlation analyses between the MDS RLU and CSF, as well as Aβ_42_ and PIB SUVR, were also conducted. Because no correlation was observed between MDS and age, a bivariate correlation analysis was performed. The ROCs were analyzed for comparing the diagnostic performance of each biomarker (MDS RLU, CSF Aβ_42_, CSF tTau/Aβ_42_, and PIB SUVR). Sensitivity and specificity were calculated using the AUC. In the correlation and ROC analyses, subjects without CSF or PIB PET results were excluded. All statistical analyses were conducted using STATA version 14.0 software (StataCorp, College Station, TX, USA).

## Results

### Patient baseline characteristics

Table 1 summarizes the baseline characteristics of patients with AD (*n* = 24) and NC (*n* = 37). There was no difference in age, sex, and years of education between the two groups. The number of apolipoprotein E (ApoE) ε4 carriers (homozygote or heterozygote) was higher in the AD group (45.8%, *n* = 11) than in the NC group (17.2%, *n* = 5). Significant differences in MMSE scores were observed between the two groups. The results of CSF biomarkers (CSF Aβ_42_, CSF tTau/Aβ_42_ ratio) and amyloid PET (PIB SUVR) from the AD group were consistent with AD characteristics and were significantly different from those of the NC group.

**Table 1:** Baseline characteristics

	NC (*n* = 37)	Patients with AD (*n* = 24)	*p* Value
Age, years	65.1 ± 7.3	67.6 ± 7.6	0.2005
Male sex	16 (43.2%)	13 (56.5%)	0.6965
Education, years	12.2 ± 3.9	13.1 ± 4.0	0.3659
MMSE score, median	29	19	<0.0001
ApoE ε4 carrier^a^	5 (17.2%)	11 (45.8%)	0.018
CSF Aβ_42_^b^, pg/ml	465 ± 117	259 ± 72	< 0.0001
CSF t-Tau/Aβ_42_ ratio^b^	0.1157	0.4184	< 0.0001
PIB SUVR^b^	1.11	1.61	< 0.0001

*Abbreviations: AD* Alzheimer’s disease, *NC* Cognitively normal control subjects, *Aβ*_*42*_ Amyloid-β 1–42 peptide, *CSF* Cerebrospinal fluid, *MMSE* Mini Mental State Examination, *ApoE* Apolipoprotein E, *PIB SUVR* ^11^C-Pittsburgh compound B standardized uptake value ratio, *pTau* Phosphorylated tau protein, *tTau* Total tau protein

^a^Twenty-four patients with AD and 29 NC were evaluated for the presence of the ApoE ε4 allele

^b^Twenty-three patients with AD and 28 NC were assessed by CSF and PIB positron emission tomography studies

### Measurement of Aβ oligomers with MDS

**Fig. 2:**
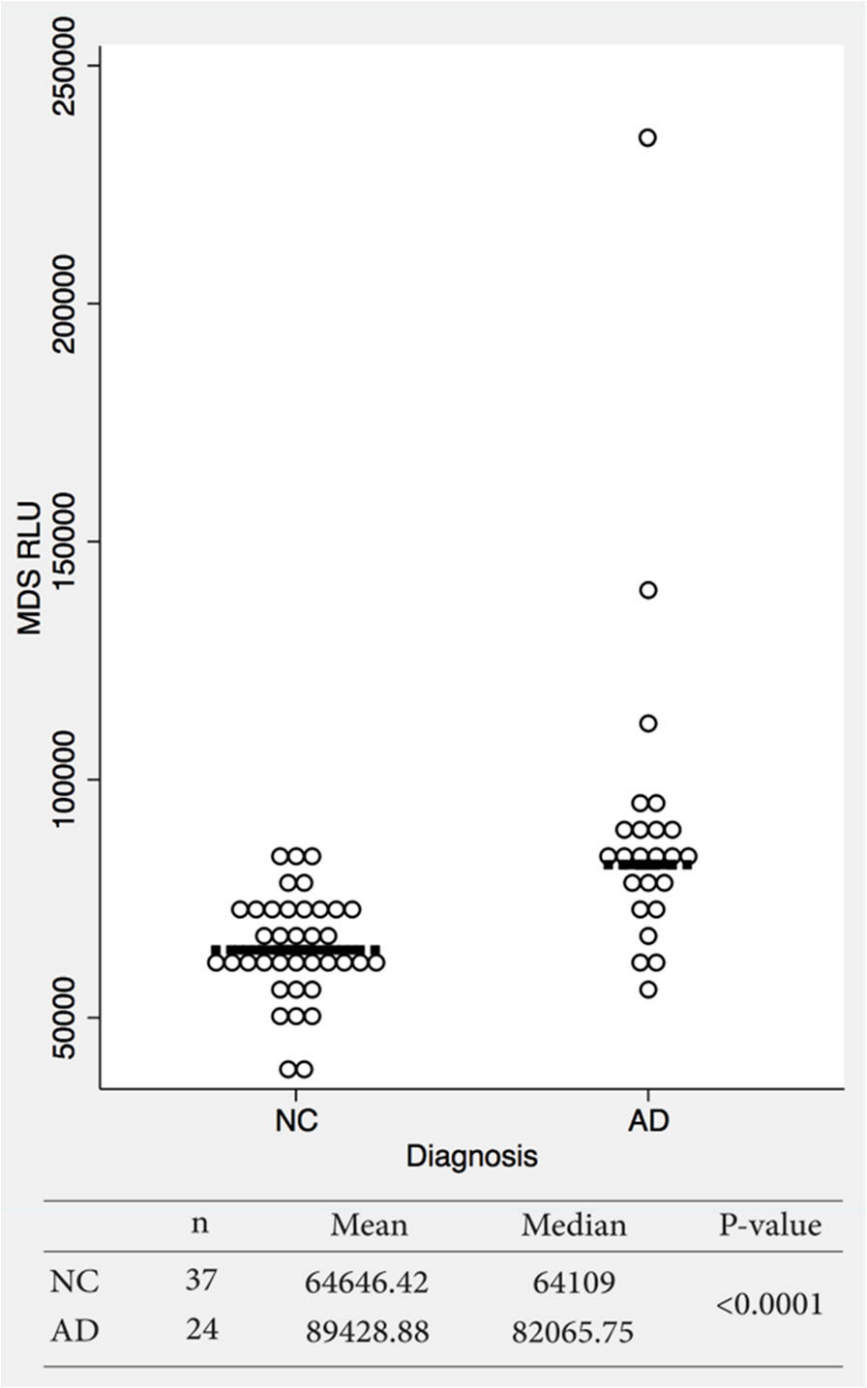
The distribution pattern of plasma amyloid-β oligomers determined using the Multimer Detection System (MDS) in patients with Alzheimer’s disease (AD) and cognitively normal control subjects (NC). The MDS relative luminescence units (RLUs) were higher in the AD group than in the NC group (*p* < 0.0001). The *horizontal bar* is the median MDS RLU. However, there was an overlap between the two groups, suggesting that further optimization of the MDS is required

**Fig. 3:**
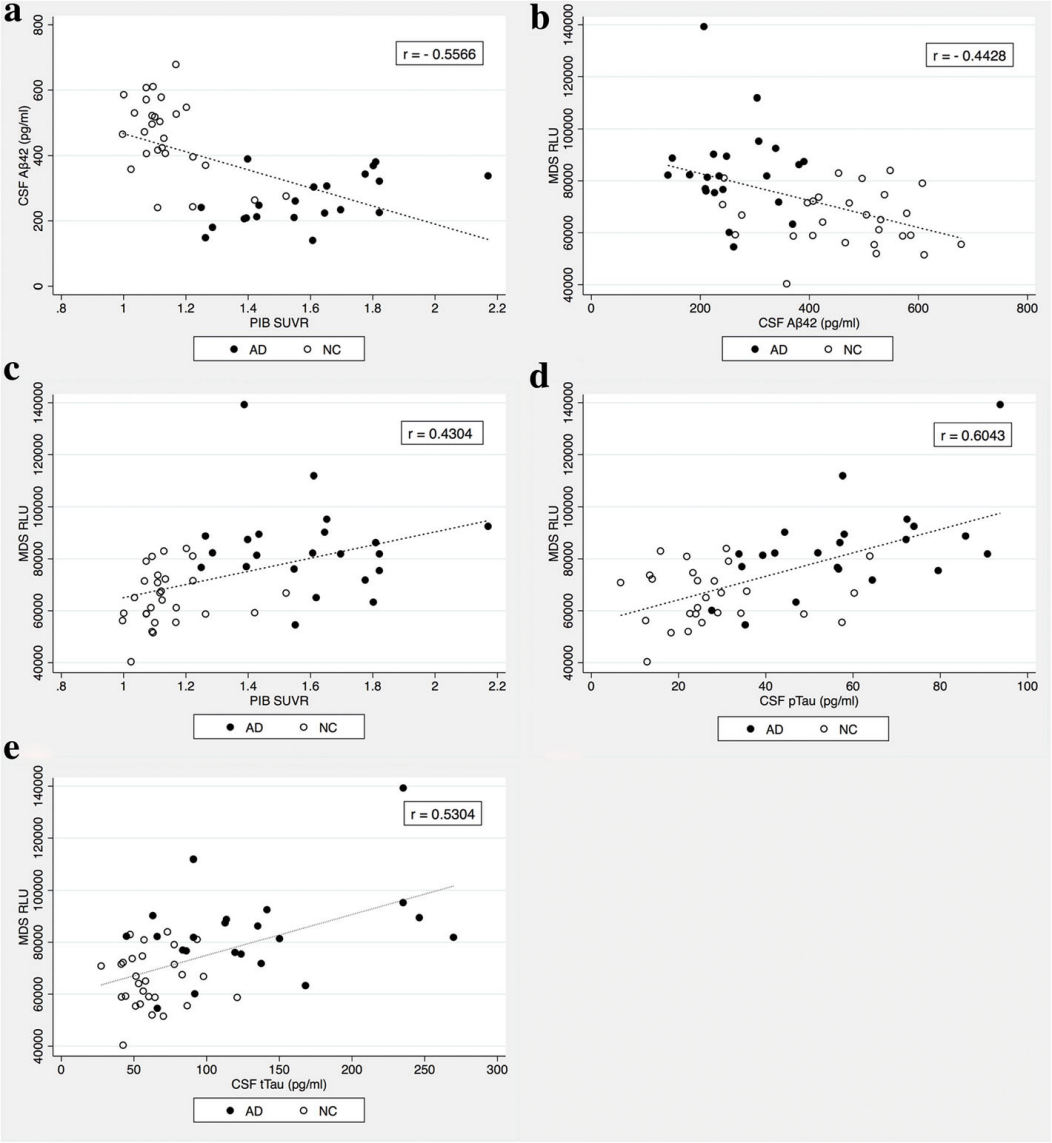
Correlations between plasma Aβ oligomer levels determined using the Multimer Detection System, and other amyloid biomarkers of Alzheimer’s disease. **a** There was a negative correlation between CSF Aβ_42_ levels and the PIB SUVR. **b** Plasma Aβ oligomer levels were moderately negatively correlated with CSF Aβ_42_ levels. **c** There was a positive correlation between plasma Aβ oligomer levels and PIB SUVR. **d** CSF pTau and **e** tTau levels also correlated positively with plasma Aβ oligomer levels. *MDS RLU* Multimer Detection System relative luminescence units, *CSF Aβ*_*42*_ Cerebrospinal fluid amyloid-β 1–42 peptide, *PIB SUVR* ^11^C-Pittsburgh compound B standardized uptake value ratio, *pTau* Phosphorylated tau protein, *tTau* Total tau protein, *AD* Patients with Alzheimer’s disease, *NC* Cognitively normal control subjects

Figure 1 depicts the rationale for using MDS in measuring Aβ oligomers in the plasma. When the Aβ oligomers were quantitated by MDS, higher oligomer levels were observed in patients with AD than in NC (Fig. 2). Subjects without CSF or PIB PET data were excluded in the correlation analysis. Thus, 51 subjects (AD, *n* = 24; NC, *n* = 29) were included in the correlation analyses. Four subjects (two in each group) without either CSF or PIB PET results were excluded from the correlation analyses between CSF biomarkers and PIB PET. Figure 3 indicates the correlations between the results of MDS and conventional AD biomarkers, including the CSF study and PIB PET. The correlation coefficient between CSF Aβ_42_ and amyloid PET was 0.5566 (Fig. 3a). The plasma levels of the Aβ oligomers from MDS correlated reciprocally with CSF Aβ_42_ levels (*r* = −0.4428) (Fig. 3b). The Aβ oligomers in the plasma and PIB PET showed a direct correlation (*r* = 0.4304) (Fig. 3c). CSF pTau and tTau also correlated positively with Aβ oligomers in plasma (*r* = 0. 5304 and 0.6043, respectively) (Fig. 3d and e).

### ROC analysis

**Fig. 4:**
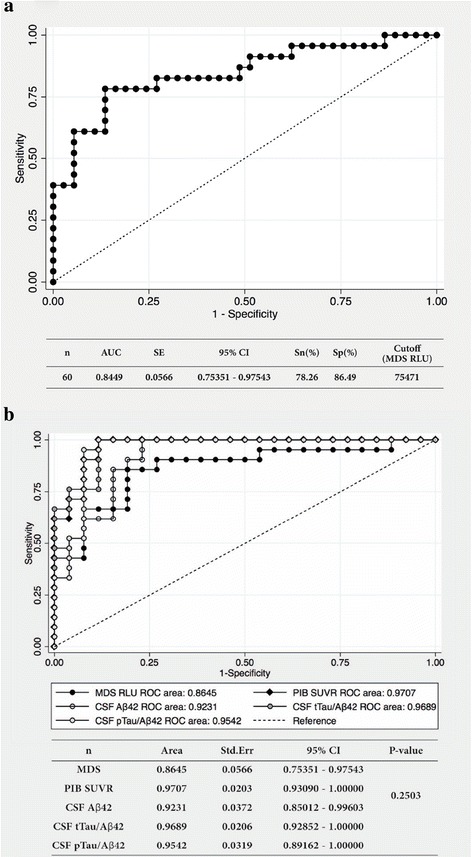
ROC analysis of plasma Aβ oligomer levels measured using the MDS. **a** ROC analysis showed that plasma Aβ oligomer levels measured using MDS could discriminate between the AD and NC groups with an AUC of 0.844. The best sensitivity and specificity were 78.3% and 86.5%, respectively. **b** The AUCs for the biomarkers were as follows: PIB SUVR (AUC 0.9707, 95% CI 0.9309–1.000), CSF tTau/Aβ42 ratio (AUC 0.9689, 95% CI 0.9285–1.000), and CSF pTau/Aβ42 ratio (AUC 0.9542, 95% CI 0.8916–1.000). The AUC for plasma Aβ oligomer levels had the lowest value (AUC 0.8645, 95% CI 0.7535–0.9754) among those of other biomarkers of Alzheimer’s disease, although the difference was not statistically significant (*p* = 0.2503). *Sn* Sensitivity, *Sp* Specificity, *MDS RLU* Multimer Detection System relative luminescence units, *PIB SUVR* ^11^C-Pittsburgh compound B standardized uptake value ratio, *CSF Aβ*_*42*_ Cerebrospinal fluid amyloid-β 1–42, *CSF tTau/Aβ*_*42*_ Cerebrospinal fluid total tau protein/Aβ_42_ ratio, *CSF pTau/Aβ*_*42*_ Cerebrospinal fluid phosphorylated tau protein/Aβ_42_ ratio

ROC analysis revealed that the AUC for plasma levels of Aβ oligomers by MDS in diagnosing AD was 0.844 (95% CI 0.7359–0.9539), suggesting a potential method for discriminating patients with AD from age-matched NC by MDS (Fig. 4a). The RLU cutoff value for the best sensitivity (78.3%) and specificity (86.5%) was 75,471 in differentiating AD from NC. Conventional AD biomarkers (PIB SUVR and the CSF tTau/Aβ_42_ ratio) also supported the above diagnostic performance of AD discrimination (PIB SUVR, AUC 0.9707, 95% CI 0.9309–1.000; CSF tTau/Aβ_42_ ratio, AUC 0.9689, 95% CI 0.9285–1.000). The AUC for plasma Aβ oligomer levels was relatively lower, whereas no significant difference was observed when compared with other biomarkers (*p* = 0.2503) (Fig. 4b).

## Discussion

In this study, an interesting ELISA technique called MDS was introduced to measure Aβ oligomers in plasma. To our knowledge, this is the first study comparing plasma Aβ oligomer levels with other conventional AD biomarkers, including CSF triple biomarkers, and amyloid PET imaging. The levels of Aβ oligomers in the plasma were higher in patients with AD than in NC. The correlation coefficient indicated a moderately strong relationship between the levels of Aβ oligomers in the plasma and other AD biomarkers. The diagnostic performance of measuring plasma levels of the Aβ oligomers by MDS was also comparable with that of other conventional AD biomarkers. Thus, we investigated the possibility of using a simple blood test to verify the presence of Aβ oligomers, which is considered a core pathological feature of AD [1]. Lesné et al. [6] identified three types of Aβ oligomers (Aβ dimers, Aβ trimers, and Aβ*56) in brain tissue. They also analyzed the correlations between the levels of Aβ oligomers and tau proteins and concluded that the presence of Aβ*56 correlated well with AD pathology. Aβ oligomers inhibited hippocampal long-term potentiation, which was related to synaptic loss and altered neuronal plasticity [7, 8]. On the basis of these results, Aβ oligomers have been investigated widely as therapeutic targets of AD.

Because the detailed basic concept of MDS for detecting Aβ oligomers was recently published [15], the present approach of measuring Aβ oligomers by MDS was expanded to a larger cohort with clinically well-characterized patient samples for the cross-comparisons in parallel with other biomarkers, such as CSF Aβ, tTau, pTau, and amyloid depositions by PIB-PET. As mentioned in the Background section, MDS required the use of epitope-overlapping antibodies against the N-terminus of Aβ for capturing and detecting the antigen in oligomeric forms. When a unique epitope on monomeric Aβ forms was bound by the capture antibody, no additional epitope would be available to the detection antibody for binding. However, Aβ oligomers with multiple copies of a unique epitope could bind both the capturing and detection antibodies, assuming free accessibility by antibodies to the binding target. In the present study, both well-characterized capturing and detection antibodies against the N-terminus Aβ were used. Therefore, the MDS would be more effective in detecting Aβ oligomers in samples than ELISAs with conformation-specific antibodies.

In previous studies, plasma levels of Aβ oligomers have been analyzed using different approaches. Zhou et al. [9] reported that increased plasma levels of Aβ oligomers detected using traditional ELISA were negatively associated with cognitive function. Another research group used sandwich ELISA [10] and reported that levels of Aβ oligomers were consistently higher in both the plasma and brain tissue of patients with AD. A combination of immunoprecipitation using magnetic beads and flow cytometry was also used for detecting plasma Aβ oligomers [11], which showed that the ROC curve analysis had a specificity of 81.2% and a sensitivity of 70.6% (AUC 0.707, 95% CI 0.52–0.853). In this study, we demonstrated that the MDS technique had a relatively higher diagnostic performance than the previous methods.

Because authors of previous meta-analyses have not found any significant difference between the total plasma Aβ_40_ and Aβ_42_ levels in patients with AD and control individuals [19–24], detecting crude oligomeric Aβ in plasma was a challenge, owing to its low concentrations in the blood, especially in the midst of several interfering factors at high concentrations. Aβ autoantibodies, albumin, fibrinogen, immunoglobulin, apolipoprotein J, ApoE, transthyretin, α-2-macroglobulin, serum amyloid P component, plasminogen, and amylin [19–23] could interfere with the detection of oligomeric Aβ by MDS. Hence, MDS for AD was optimized to enhance detection by spiking synthetic Aβ_42_ into the plasma as mentioned in a recent study [15]. Therefore, in the present study, we used the optimized approach as well.

The aim of the present study was to verify whether the levels of Aβ oligomers in plasma by MDS correlated well with other AD biomarkers. The levels of Aβ oligomers in the plasma by MDS were higher in the AD group than in the NC group, consistent with previous reports [9–11]. We confirmed that the correlation of the plasma levels of Aβ oligomers with other conventional amyloid biomarkers was similar to that of CSF Aβ_42_ and PIB SUVR with moderately strong correlations. The present study might be the first to present a direct correlation between levels of Aβ oligomers in plasma and other amyloid biomarkers. Most studies thus far have shown no or low correlations between plasma Aβ_40_ or Aβ_42_ and CSF Aβ_42_ or PET amyloid plaques [25, 26]. However, the present results reveal that plasma levels of Aβ oligomers had a substantial correlation with CSF tau proteins, especially pTau. Previous studies have shown that levels of soluble Aβ oligomers correlated with the extent of synaptic loss and severity of cognitive impairment [7, 8], as well as that levels of tTau and pTau in CSF correlated with increased disease severity and disease progression [27, 28]. Overall, measuring the plasma levels of Aβ oligomers using the MDS technique could be associated with symptom severity, which required further investigation for its potential use in monitoring disease progression or as a prognostic biomarker of AD. The correlation analysis between plasma Aβ oligomer levels obtained using MDS and other amyloid biomarkers showed a low correlation coefficient between MDS and PIB SUVR (Additional file 1: Figure S1). This result pertained to a patient who was evaluated for AD using an amyloid biomarker mismatch (Additional file 1: Table S1 and Figure S2). Hence, few such unusual cases were excluded in the correlation and ROC analyses.

According to published AD criteria [29], a biomarker should possess > 80% sensitivity and specificity. The plasma levels of the Aβ oligomer detected using the MDS technique did not satisfy these criteria, owing to lower sensitivity. However, the specificity exceeds 80%, and the sensitivity was close to this percentage. Initially, CSF Aβ_42_ and tau protein also showed similar lower levels of sensitivity and specificity [30, 31]; however, their levels increased as the performance of the analytical protocols improved [18, 32]. Similarly, the diagnostic value of plasma Aβ oligomers for AD will increase as the MDS technique is further refined in the future.

The present study has several limitations. First, the study samples were small, owing to difficulties in obtaining willing participants for the CSF study, especially in the NC group. Because the well-characterized patients with AD were selected by experts, more reliable data on AD biomarkers were collected. The results of the present study could be supported by a larger cohort and longitudinal studies. Second, individuals with various conditions, such as mild cognitive impairment, preclinical AD, Parkinson’s disease, frontotemporal dementia, or vascular dementia, could not be included. Comparison with control samples from such diseases would help evaluate the usefulness of Aβ oligomers in the plasma as a diagnostic biomarker of AD. Third, spiking synthetic Aβ_42_ peptide into the plasma from patients with AD and NC with a long incubation time of 144 h hastened the amplification of Aβ oligomers in the plasma of patients with AD but not in the plasma of NC [15]. Moreover, the long incubation period of 144 h was a limitation of the present MDS protocol, which should be shortened drastically before clinical application. Finally, the MDS technique should be further optimized for better discrimination between AD and the control group. Although the sensitivity and specificity of MDS were less than optimal, the above factors should be considered when making efforts to improve the MDS technique during the refining process. After the optimization of MDS, further studies should be performed to reexamine whether the estimation of Aβ oligomers in the plasma could improve AD diagnosis in comparison to other biomarkers.

## Conclusions

The measurement of Aβ oligomers in the plasma by MDS might be a reliable method for diagnosing AD-associated amyloid pathology. Before adapting MDS in clinical settings, further investigations should be conducted to validate the level of Aβ oligomers in the plasma and its possibilities in screening patients, monitoring longitudinal changes throughout the course of AD, or determining the efficacy of Aβ-targeting drugs after administration. Nevertheless, assaying Aβ oligomers in the plasma would hold significant promise as a noninvasive, simple-to-perform, and inexpensive approach that might be used in routine clinical practice for detecting Aβ oligomers related to AD pathology.

## Additional file

Additional file 1: Table S1. Neuropsychological test results of a 74-year-old patient diagnosed with Alzheimer’s disease and an amyloid biomarker mismatch. **Figure S1.** Correlation between plasma Aβ oligomer levels measured using the multimer detection system and other amyloid biomarkers of Alzheimer’s disease. **Figure S2.** Brain magnetic resonance imaging and fludeoxyglucose positron emission tomography study of a 74-year-old patient diagnosed with Alzheimer’s disease and an amyloid biomarker mismatch. (DOCX 4327 kb)

## Abbreviations

AD: Alzheimer’s disease
ApoE: Apolipoprotein E CDR, Clinical Dementia Rating
Aβ: Amyloid-β
Aβ_42_: Amyloid-β 1–42 peptide
CSF: Cerebrospinal fluid
ELISA: Enzyme-linked immunosorbent assay
HRP: Horseradish peroxidase
MDS: Multimer Detection System
MMSE: Mini Mental State Examination
MRI: Magnetic resonance imaging
NC: Normal controls
PBST: PBS containing Tween-20
PET: Positron emission tomography
PIB: ^11^C-Pittsburgh compound B
pTau: Phosphorylated tau protein
RLU: Relative light/luminescence units
SUV: Standardized uptake value
SUVR: Standardized uptake value ratio
TBST: Tris-buffered saline with Tween 20
tTau: Total tau protein
