# Supplementary material for: Oligomeric forms of amyloid-β protein in plasma as a potential blood-based biomarker for Alzheimer’s disease
Source: Alzheimers Res Ther. 2017 Dec 15;9:98. doi: 10.1186/s13195-017-0324-0 (PMC5732503; doi:10.1186/s13195-017-0324-0)
Supplement: Additional file 1: Table S1. — Neuropsychological test results of a 74-year-old patient diagnosed with Alzheimer’s disease and an amyloid biomarker mismatch. Figure S1. Correlation between plasma Aβ oligomer levels measured using the multimer detection system and other amyloid biomarkers of Alzheimer’s disease. Figure S2. Brain magnetic resonance imaging and fludeoxyglucose positron emission tomography study of a 74-year-old patient diagnosed with Alzheimer’s disease and an amyloid biomarker mismatch. (DOCX 4327 kb) [file 13195_2017_324_MOESM1_ESM.docx]

**Additional files**

| **Table S1** Neuropsychological test results of a 74-year-old patient with Alzheimer’s disease and an amyloid biomarker mismatch | | | |
| --- | --- | --- | --- |
| **Tests** | | **Raw score** | **Percentile score** |
| **Boston Naming Test** | | 7 | 0.01 |
| **SVLT** | **Immediate recall** | 7 | 0.01 |
|  | **Delayed recall** | 0 | 0.33 |
|  | **Discrimination index** | 5 | 5.16 |
| **RCFT** | **Copy** | 23.5 | 0.02 |
| **SWF** | **Animal** | 10 | 3.07 |
|  | **Alphabet** | 4 | 9.51 |
| **Stroop test** | **Word reading** | 108 | <16 |
|  | **Color reading** | 45 | 0.12 |
| **MMSE** | | 19 | - |
| **CDR** | | 1.0 | - |
| **CDR Sum of Box** | | 5.5 | - |
| **Short form GDpS** | | 0 | - |
| SVLT: Seoul verbal learning test; RCFT: Rey copy figure test; SWF: Semantic word fluency; MMSE: Mini-mental screening test; CDR: Clinical dementia rating; GDpS: Geriatric depression score. | | | |

**Figure S1.** Correlation between plasma Aβ oligomer levels measured using the multimer detection system and other amyloid biomarkers of Alzheimer’s disease

**
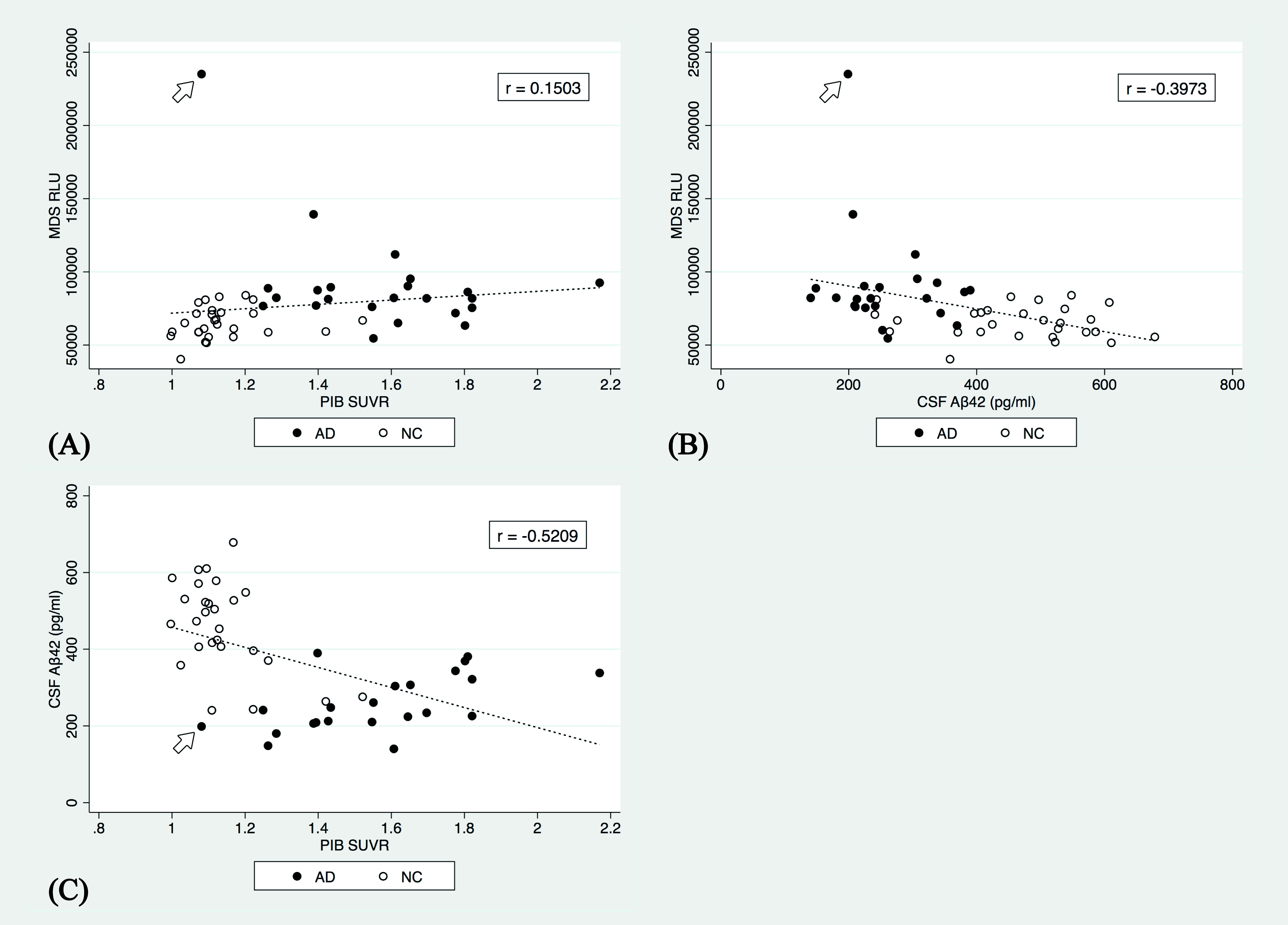
**

Correlations between plasma Aβ oligomer levels measured using the MDS and other amyloid biomarkers were determined for 23 Alzheimer’s disease and 28 normal control subjects who underwent both the cerebrospinal fluid study and ^11^C-Pittsburgh compound B positron emission tomography. (A) The correlation coefficient between MDS and PIB SUVR was low (r = 0.1503). (B) Plasma Aβ oligomer levels and CSF Aβ_42_, and (C) CSF Aβ_42_ and PIB PET, were negatively correlated. These results were from a patient evaluated to have Alzheimer’s disease with an amyloid biomarker mismatch (open arrow). The 74-year-old patient showed high levels of Aβ oligomers by MDS (235,108 RLU) and a low CSF Aβ42 value (198.7 pg/mL), that were compatible with Alzheimer’s disease, but with a low PIB SUVR (1.08).

MDS RLU: Multimer detection system relative luminescence units; CSF Aβ42: Cerebrospinal fluid amyloid beta 1-42; PIB SUVR: ^11^C-Pittsburgh compound B standardized uptake value ratio

**Figure S2.** Brain magnetic resonance imaging and fludeoxyglucose positron emission tomography study of a 74-year-old patient evaluated to have Alzheimer’s disease and an amyloid biomarker mismatch


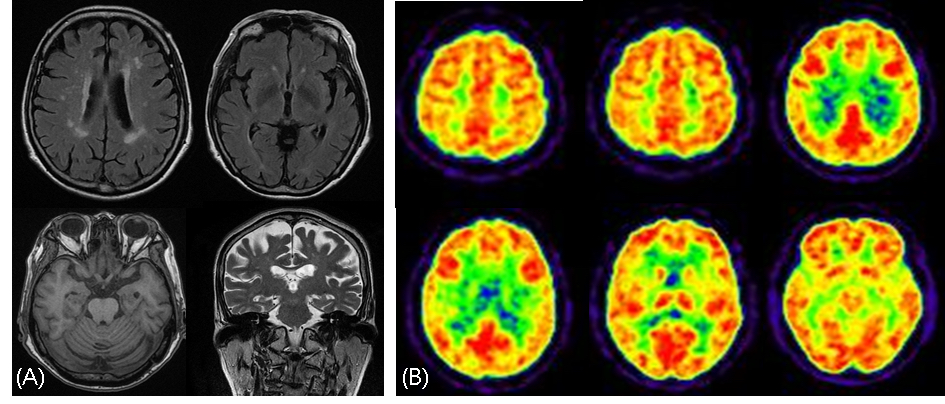


The patient showed bilateral hippocampal atrophy (right = grade 1, left = grade 2 by Shelton’s criteria (Scheltens et al., 1995)) and small vessel disease on brain magnetic resonance imaging. Fludeoxyglucose positron emission tomography revealed hypometabolism in the bilateral parietal lobes.
